# Effect of Silane Coupling Agent Modification on Properties of Brass Powder-Water-Based Acrylic Coating on *Tilia europaea*

**DOI:** 10.3390/polym15061396

**Published:** 2023-03-10

**Authors:** Yan Han, Xiaoxing Yan

**Affiliations:** 1 Co-Innovation Center of Efficient Processing and Utilization of Forest Resources, Nanjing Forestry University, Nanjing 210037, China; 2 College of Furnishings and Industrial Design, Nanjing Forestry University, Nanjing 210037, China

**Keywords:** art coating, brass powder, optical properties, silane coupling agent, mechanical properties, liquid resistance, aging resistance

## Abstract

Fine art coating is usually created by the combination of metal filler and water-based coatings, decorated to the surface of wood structures, furniture, and crafts. However, the durability of the fine art coating is limited by its weak mechanical qualities. In contrast, the metal filler’s dispersion and the coating’s mechanical properties can be significantly improved by the coupling agent molecule’s ability to bind the resin matrix with the metal filler. In this study, a brass powder-water-based acrylic coating was prepared, and three different silane coupling agents, 3-aminopropyltriethoxysilane (KH550), γ-(2,3-epoxypropoxy)propytrimethoxysilane (KH560), and γ-methacryloxypropyltrimethoxysilane (KH570), were used to modify the brass powder filler in orthogonal tests. The artistic effect and optical properties of the modified art coating induced by different proportions of brass powder, silane coupling agents, and pH were compared. The result demonstrated that the amount of brass powder and the kind of coupling agent used had a substantial impact on the coating’s optical characteristics. Our results also determined how three different coupling agents affected the water-based coating with varying brass powder contents. The findings indicated that 6% KH570 concentration and pH 5.0 were the ideal conditions for brass powder modification. Better overall performance of the art coating applied to the surface of the Basswood substrates was provided by adding 10% of the modified brass powder into the finish. It had a gloss of 20.0 GU, a color difference of 3.12, a color main wavelength of 590 nm, a hardness of HB, an impact resistance of 4 kg·cm, an adhesion of grade 1, and better liquid resistance and aging resistance. This technical foundation for the creation of wood art coatings promotes the application of art coatings on wood.

## 1. Introduction

Art coating usually serves a decorative effect [1,2,3] but also has more robust protection than general coatings [4,5]. Studies [6,7,8] have shown that adding metal filler in the water-based coating and applying a modified art coating can lend the surface of buildings, furniture, and handicrafts a shiny metallic luster. Consequently, this modified coating provides a protective and decorative role to the applied products, causing an increase in added value [9]. Therefore, modified art coatings have a valued decorative material in the decorating industry, with a large annual consumption [10].

In coatings market segments like architecture, product finishes and specialty coatings consisting of acrylic resins are frequently used. The three primary categories of acrylic coatings are water-based acrylic coatings [11], solvent-based acrylic coatings [12], and acrylic powder coatings [13]. Water-based acrylic resin has a wide range of application possibilities for metal powder coating due to its superior hardenability, smooth finish, excellent stain resistance, fewer VOCs emission, low cost, and simple preparation [14,15,16]. The major issues are unavoidable shortcomings, such as being sticky when hot and brittle when cold and having poor anti-return adhesion, and inadequate thermal stability after drying into a film [17,18]. Kim et al. [19] successfully synthesized multifunctional acrylic polyurethane coating materials with graffiti, pollution, and adhesive resistance qualities by using low-viscosity single-ended silicone oil and acrylic polyols with high glass transition temperatures as raw materials. In order to increase the water resistance of the resin coating, Yu et al. [20] effectively synthesized water-based acrylic resin modified by itaconic acid and gamma-methacryloxypropyl triisopropoxidesilane by seeded emulsion polymerization.

Furthermore, a growing body of evidence [21,22,23] indicates that the requirement from consumers for a decorative appearance is increasing. Therefore, the demand for eco-friendly, individualized coatings, with low consumption, has turned into a development trend in fine art coating technology. Adding metal powder as filler into water-based coatings can achieve these goals. However, due to the poor compatibility with water-based coatings, pure metal powder filler is easily agglomerated and dispersed unevenly within the coating, which affects the optical properties and worsens the mechanical properties, making them brittle, prone to cracking, and less adhesive [24]. These deficiencies severely restrict their practical application and advancement.

The coupling agent, a kind of additive, has the ability to enhance the bonding performance between inorganic and organic substances [25]. Due to the fact that its structure contains two different properties of groups, one of which is pro-inorganic or easily reacts with the surface of inorganic materials, while the other is pro-organic or can cause reactions with organic polymers physico-chemically. Therefore, coupling agents, or “molecular bridges”, could reinforce the interaction between organic and inorganic molecules, considerably enhancing the performance of composite materials. Currently, the coupling agents most frequently employed are silane-based. A silane-based coupling agent’s general formula is Y-R-Si-X_3_, where Y stands for the organic functional group and R for the alkylidene group, and X represents the group that can be hydrolyzed. Thus, the application of coupling agent molecules to metal powder coatings could open the door for their practical use. The adhesion behavior of polyurethane (PU), polyurethane acrylate (PUA), and two coats loaded with silicone rubber (SR) nanoparticles on tin pads was examined by Sivakumar et al. [26] in relation to the use of silane agents. On tin surfaces, it was discovered that silanes based on isocyanates offered outstanding adhesion for PU and PU + SR coatings, while silanes based on acrylic offered acceptable adhesion for PUA and PUA + SR coatings. For polyvinyl butyral coatings, Zhu et al. [27] used reduced graphene oxide and TiO_2_-KH550 fillers to increase the corrosion resistance of 304 stainless steel in various conditions. Li et al. [28] produced antioxidant functionalized silica-coated TiO_2_ nanorods utilizing KH550 as a bridge in a successful synthetic process. By means of melt mixing, the AO-KH550-SiO_2_-TiO_2_ was added to the polypropylene matrix. According to the findings, AO-KH550-SiO_2_-TiO_2_ might enhance polypropylene’s photostability and thermal stability. Wang et al. [29] coated the surface of B4C particles with KH560 in order to accomplish the optimal dispersion of particles in the epoxy coating and increase the chemical interaction between particles and polymer coating. In addition to enhancing the interaction between B4C particles and epoxy resin, KH560 also demonstrates the capacity to greatly enhance epoxy resin’s anti-corrosion performance. By using a unique silane coupling agent sensitization approach that is based on three distinct functional groups of silane coupling agents (KH550, KH570, and KH792), Zou et al. [30] examined the mechanism of chemical copper plating of intermediate-phase asphalt-based carbon fibers. In the copper-plated carbon fibers modified by KH550 grafting, larger grain size, reduced resistivity, and more uniform and continuous copper coating were seen.

Brass powders are inexpensive, easily accessible, and have vibrant, sparkling colors that are incredibly ornamental. There are, however, few studies on coating wood surfaces with water-based coatings that contain brass powder that has been treated with silane coupling agents. Coatings containing pristine brass powder have weak mechanical characteristics, poor age resistance, and poor liquid resistance, making it challenging for them to meet the standards of commercial coatings for wood surfaces [31,32]. The purpose of this paper is to modify copper powder by three different types of silane coupling agents, screen the suitable coupling agent type, optimize the copper powder modification process, and obtain the metal powder wood coatings with the best mechanical properties, liquid resistance, aging resistance, and, at the same time, good optical properties.

In the present study, brass powder was selected as a filler and three types of silane coupling agents (KH550, KH560, KH570) were used for modification to prepare brass powder-water-based acrylic coatings. An orthogonal test based on the optical properties was carried out to investigate the effects of four factors, coupling agent type, coupling agent content, pH value at modification, and brass powder content, on the coating’s gloss, color difference, and chromaticity variation. After identifying the most significant influencing elements, single-factor experiments were designed to assess the optical, physical, dry-heat aging, ultraviolet (UV) photooxidation, and cold liquid resistance qualities of the films. These results establish the optimum method for preparing brass powder-water-based acrylic coating and provide technical evidence for the creation of fine art coatings.

## 2. Materials and Methods

### 2.1. Test Materials

Brass powder (size 1000 mesh, diameter about 13 µm) was provided by Nangong Xindun Alloy Welding Material Spraying Co., Ltd., Xingtai, China. 3-aminopropyltriethoxysilane (KH550, C_9_H_23_NO_3_Si, M_w_: 221.37 g/mol, CAS No.: 919-30-2), γ-(2,3-epoxypropoxy)propytrimethoxysilane (KH560, C_9_H_20_O_5_Si, M_w_: 236.36 g/mol, CAS No.: 2530-83-8), γ-methacryloxypropyltrimethoxysilane (KH570, C_10_H_20_O_5_Si, M_w_: 248.35 g/mol, CAS No.: 2530-85-0) were provided by Hangzhou Jessica Chemicals Co., Ltd., Hangzhou, China. Dulux water-based acrylic primer and topcoat were provided by Dulux Coatings Co., Ltd., Shanghai, China. Citric acid monohydrate (C_6_H_10_O_8_, M_w_: 210.14 g/mol, CAS No.: 5949-29-1) was acquired from Suzhou Changjiu Chemical Technology Co., Ltd., Suzhou, China. Anhydrous ethanol (C_2_H_6_O, M_w_: 46.07 g/mol, CAS No.: 64-17-5) was acquired from Wuxi Jingke Chemical Co., Ltd., Wuxi, China. *Tilia europaea* (Basswood) boards (100 × 50 × 5 mm^3^) were purchased from Beijing Yidimei Model Co., Ltd., Beijing, China. Detergent was obtained from Lishui Diaopai Chemical Co., Ltd., Lishui, China. Coffee was obtained from UCC Ueshima Coffee Co., Ltd., Kobe, Japan.

### 2.2. Silane Modification Method of Brass Powder

A water/alcohol mixture was prepared as a solvent with a mass ratio of ethanol-to-water of 4:1 to carry out the silane hydrolysis reaction [33], and the pH value was adjusted with citric acid monohydrate [34]. A certain quantity of silane coupling agent was weighed according to the different silane coupling agent concentrations (m _silane coupling agent_: m _brass powder_), put into the prepared water/alcohol mixture, and stirred at room temperature until it was fully hydrolyzed to obtain the silane modification solution. The brass powder was then added to the silane-modified solution and stirred for 3 h at a speed of 600 rpm in a DF-101s thermostatic heating magnetic stirrer (Gongyi Yingyu Instrument Co., Ltd., Gongyi, China) at 30 °C. After the reaction was terminated, the obtained material was rinsed and filtered repeatedly with anhydrous ethanol and deionized water, respectively, by an SHZ-D(III) circulating water multi-purpose vacuum pump (Zhengzhou Keda Machinery Instrument Equipment Co., Ltd., Zhengzhou, China) to remove the unreacted silane modifier, and then was put into a 60 °C DYJG-9023 precision blast drying oven (Hangzhou Yijie Technology Co., Ltd., Hangzhou, China) to obtain the modified brass powder. Table 1 shows the dosage of modified brass powder with different silane coupling agent contents.

### 2.3. Preparation of Water-Based Acrylic Coating with Brass Powder

**Figure 1:**
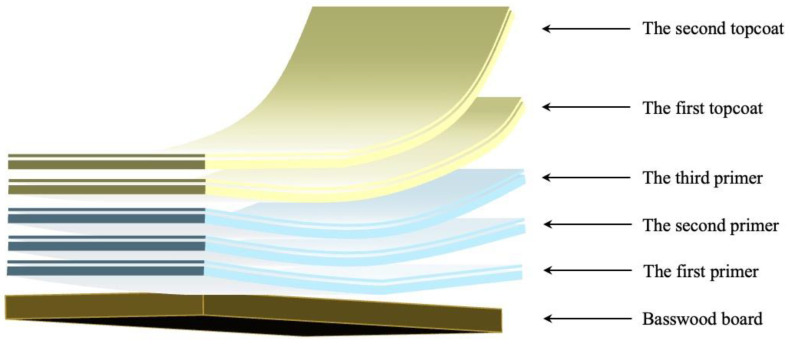
The structure of the prepared brass powder water-based acrylic coating.

Firstly, the 2.0 g of water-based primer was weighed and evenly applied to the surface of the Basswood substrate three times. After each application, the sample was exposed to air for 20 min before being cured and dried in an oven at 60 °C. After each coat of primer is finished, it was sanded with 1000 mesh sandpaper. Next, the modified brass powder was added to the water-based acrylic topcoat at a certain mass concentration and mixed well with a glass rod to obtain a 2.0 g coating as the topcoat. The prepared topcoat coating was evenly applied on top of the primer film with the third layer two times, exposed to air for 20 min, and cured in a 60 °C oven until the quality no longer changed. Figure 1 depicts the structure of the prepared brass powder water-based acrylic coating.

### 2.4. Orthogonal Experiment Design

A silane-based coupling agent can improve the metal filler’s oxidation resistance and aging resistance in the coating system because of its wettability and dispersion with the metal filler. Common silane coupling agents used in metal-resin systems include KH550, KH560, KH570, etc. The concentration of the silane coupling agent, in addition to the type, has a significant impact on the coating effect with metal filler [35]. Meanwhile, the silane solution’s pH influences the rate of the reaction between polycondensation and hydrolysis, which in turn impacts how much film forms on the surface of the metal filler [36]. While silane can hydrolyze more quickly in both acidic and alkaline settings, condensation of silane occurs more readily in an alkaline environment. In order to prevent significant amounts of silane condensation and flocculation during the reaction, the pH is often fixed between 3 and 5.

Consequently, the aforementioned variables were used to develop a four-factor, three-level orthogonal experiment of L_9_(3^4^), with the following four factors: pH value, copper powder content, silane coupling agent type, and silane coupling agent content, according to Table 2 and Table 3. The dosage details of the coating system of the orthogonal test samples are shown in Table 4.

### 2.5. Single-Factor Experiment Design

The prepared paint film samples were subjected to orthogonal analysis in terms of gloss, color difference, and chromaticity variation to determine the maximum influencing factors. The experimental process was optimized for its maximum influencing factor to investigate the effect of modifications by different silane coupling agents on the performance of brass powder water-based acrylic coatings. The materials and preparation process were kept unchanged, the concentration of the silane coupling agent was fixed at 6.0% and the pH value at modification was 5.0. The effect of brass powder modified by three different silane coupling agents (KH550, KH560, and KH570), and the content of unmodified brass powder on the overall qualities of the film was investigated (Table 5).

### 2.6. Testing and Characterization

#### 2.6.1. Characterization of Micro-Morphology and Chemical Composition

The appearance of treated and untreated brass powder was observed using an EM Crafts VERITAS scanning electron microscope (SEM, Korea EM Crafts, Seoul, Korea). Chemical composition analysis of treated brass powder, untreated brass powder, and film samples was performed by a BOEN-85697 Fourier transform infrared spectrometer (FTIR, Fairborn Precision Instruments Co., Ltd., Shanghai, China).

#### 2.6.2. Optical Performance Testing

According to the standard GB/T4893.6-2006 [37], the gloss of the coating was evaluated using a 3NH touchscreen triangle glossmeter NHG268 (Guangzhou Threenh Technology Co., Ltd., Guangzhou, China), which was utilized to measure the gloss at different incidence angles (20°, 60°, and 85°).

A Kutai MS3003 multi-angle spectrophotometer (Guangzhou Threenh Technology Co., Ltd., Guangzhou, China) was utilized to measure the color difference of the coating, and three groups of data *L*_1_, *a*_1_, and *b*_1_ were outputted by the colorimeter at a random location on the film, and another group of data *L*_2_, *a*_2_, and *b*_2_ were measured by the colorimeter at another location on the film. According to the CIE94 color difference Formula (1), Δ*L* is the difference between *L*_1_ and *L*_2_, Δ*a* (red/green difference) is the difference between *a*_1_ and *a*_2_, and Δ*b* is the difference between *b*_1_ and *b*_2_, to calculate the color difference value (Δ*E*) of the film [38,39].

(1) $$\Delta E\;=\;\sqrt{{\left(\Delta L\right)}^{2}\;+\;{\left(\Delta a\right)}^{2}\;+\;{\left(\Delta b\right)}^{2}}$$

In addition, the colorimetric values of water-based paint films with 10%, 20%, 30%, 40%, 50%, and 60% brass powder content were measured using a SEGT-J portable colorimeter, and four points were taken for each film, and the means were calculated as the colorimetric values of the film, which were recorded as L*, a*, and b*. Three colorimetric parameters were subtracted from the colorimetric values of pure water-based paint films without brass powder to obtain ΔL*, Δa*, Δb*. The chromaticity variation of the film with different brass powder content was estimated according to Formula (1) and noted as ΔE* [40,41,42].

The wavelength-reflectance curves, color dominant wavelengths, and color saturation of the Basswood board surface coatings in the visible wavelength range (380–780 nm) were obtained using a Hitachi Model U-3900/U-3900H UV spectrophotometer (Hitachi Limited, Tokyo, Japan) with a scanning speed of 600 nm/min. According to ASTM G173-03 [43], Formula (2) was used to calculate the solar reflectance (*R*) of the coating in the visible range, where *i*(*λ*) is the standard solar radiation intensity and *r*(*λ*) is the reflectance value obtained after testing.

(2) $$R\;=\;\frac{\int_{380}^{780}r\left(\lambda \right)i\left(\lambda \right)d\left(\lambda \right)}{\int_{380}^{780}i\left(\lambda \right)d\left(\lambda \right)}$$

#### 2.6.3. Mechanical Performance Testing

According to the standard GB/T6739-2006 [44], a QHQ-A portable pencil hardness tester (Liangchuang Instrument Co., Ltd., Suzhou, China) was utilized to fix a pencil (6B–6H) and pressed down on the surface of the film at 45° under a load of 750 g, gradually increasing the pencil hardness until the film had a lasting depression, at which time the pencil’s hardness was recorded as the coating’s hardness.

According to the standard GB/T4893.9-2013 [45], a BEVS 1601 impact tester (Beijing Shidai Shanfeng Technology Co., Ltd., Beijing, China) was utilized to assess the coating’s impact resistance. The painted wooden board was put on the impact tester with the steel ball directly above the tested surface, then the steel ball was lifted to a certain height and allowed to free-fall impact on the board. The lowest height for the coating to be broken was used to calculate the impact resistance strength.

According to the standard GB/T4893.4-2013 [46], a QFH-HG600 hundred grid knife (Dongguan Zhongte Precision Instrument Technology Co., Ltd., Dongguan, China) was utilized to assess the coating’s adhesion, using a cutting tool to apply evenly force perpendicular to the coating surface to form a set of parallel cutting lines on the coating, and then repeating the above operation to cut on the coating surface and intersecting with the original cutting line at 90° to form a grid pattern. Then the whole grid was covered with tape for 5 min. After smoothly tearing off the tape, the level of coating adhesion was determined with a total of 1–5 levels, with level 1 being the best.

Using the JB-6C roughness tester (Shanghai Gaozhi Precision Instrument Co., Ltd., Shanghai, China), the coated Basswood board was put on the testing table, and the probe was moved to make contact with it. The probe position was adjusted to make it stable at the 0 coordinate, and then the roughness R_a_ was recorded.

#### 2.6.4. Test of Aging Resistance of the Film

The film samples were subjected to accelerated aging at 160 °C in an oven to carry out dry-heat aging resistance testing [47]. The UV photooxidation resistance testing was performed on the prepared paint samples by using a UVA-340 fluorescent lamp according to ASTM D4587-2011 [48] with a cycle of “4 h irradiation (0.89 W·m^−2^·nm^−1^) and 20 h condensation” three times. The coating’s chromaticity variation ΔE* was calculated by Formula (1) to evaluate its aging resistance.

#### 2.6.5. Test of Cold Liquids Resistance of the Film

According to the standard GB/T4893.1-2021 [49], citric acid solution with a mass fraction of 10%, undenatured ethanol with a volume fraction of 96%, detergent, and coffee (freeze-dried 40 g instant coffee in 1 L boiling water) were selected as test solutions. Before starting the experiment, the coating’s surface was gently wiped with a clean cloth, and a soft filter paper of 25 mm diameter without stain and adhesive was soaked in the test solution, removed, and placed on the surface of the coating, which was then placed in an airtight environment. After a certain period of time, the leftover liquid on the surface was discarded after the filter paper was removed, and the damage to the surface of the coating was observed after standing for 24 h, and the cold liquid resistance grade was evaluated with reference to Table 6. Before and after the liquid resistance test, the surface coating on the Basswood boards was measured for chromaticity using a color difference meter, and the chromaticity variation ΔE* of cold liquid resistance of the film was computed using Formula (1).

With an error of less than 5%, each of the aforementioned tests was performed four times.

## 3. Results and Discussion

### 3.1. Microscopic Morphology and Infrared Spectral Analysis of the Modified Brass Powder

**Figure 2:**
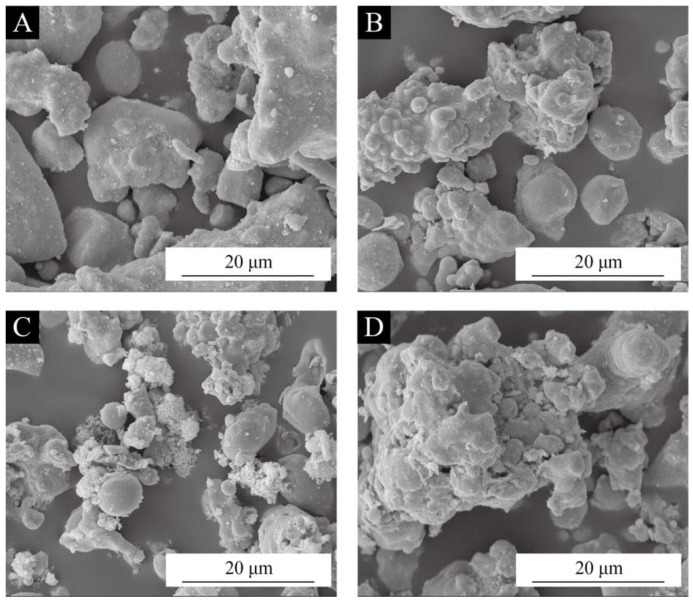
SEM images of brass powder modified by different silane coupling agents under the optimal conditions of silane coupling agent content of 6.0% and pH of 5.0: (**A**) unmodified, (**B**) KH550 modified, (**C**) KH560 modified, (**D**) KH570 modified.

To observe the encapsulating effect of the brass powder, SEM analysis was performed on the raw brass powder without silane coupling agent modification and the brass powder was modified under the optimal conditions of silane coupling agent content of 6.0% and at a pH of 5.0. From Figure 2A, the surface of the raw brass powder is flat and free of adhesion, while the brass powder’s surface, which was modified by the silane coupling agent (Figure 2B–D) is covered with a dense film; the surface is not as smooth as that of the raw brass powder and a more obvious agglomeration phenomenon appears, demonstrating the effectiveness of the coating.

**Figure 3:**
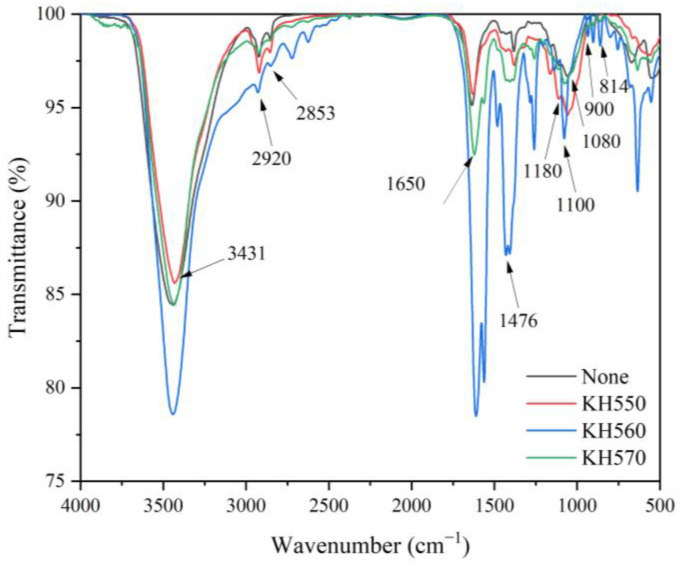
FTIR images of brass powder modified by different silane coupling agents.

Figure 3 shows the infrared spectra of brass powder modified by different silane coupling agents. For the three spectra, the characteristic peaks generated by the stretching vibration of Si-OH can be observed at 1100 cm^−1^, which is due to the conversion of the -SiOC_2_H_5_ group in KH550 and the −SiOC_2_H_3_ group in KH560 and KH570 to Si-OH during the hydrolysis process [50]. The stretching vibration peak of C-H in C-CH_3_ has a peak at 2920 cm^−1^. At 2853 cm^−1^, there is the characteristic peak of =CH_2_. The stretching vibration peak of C-C is at 900 cm^−1^. The peak at 1476 cm^−1^ belongs to the deformation vibrations of =CH_2_ and =CH_3_ [51]. The stretching vibration peak of Si-O occurs at 814 cm^−1^. The Si-O-C vibration is responsible for the peak at 1180 cm^−1^ [52]. There is a distinctive peak of the stretching vibration of the amino group in KH550 at 3431 cm^−1^ [53]. The C=O in KH570 is represented by the peak at 1650 cm^−1^ [54]. The identified peaks indicate that the brass powder’s surface has been successfully covered with the silane coupling agent.

### 3.2. Analysis of Orthogonal Test Results

#### 3.2.1. Gloss Analysis

**Figure 4:**
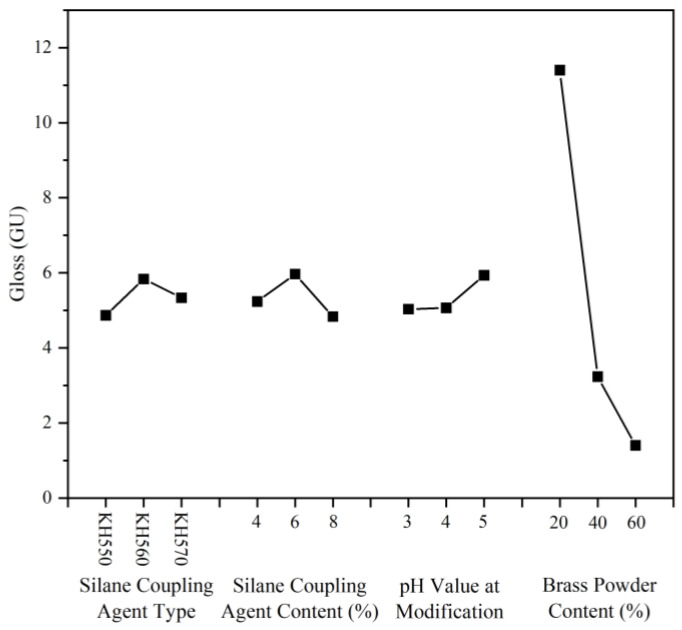
The graph of the film’s gloss effect at a 60° incidence angle.

Gloss refers to the coating surface’s capacity to specularly reflect light and serves as the foundation for determining how smooth the coating surface is. The findings are displayed in Table 7, where the gloss performance of the paint film surface is significantly indicated by the gloss corresponding to an incidence angle of 60° [55]. The visual analysis of the paint film’s gloss at a 60° incidence angle is shown in Table 8. Sample 5# had the highest gloss level of 13.1 GU, followed by sample 9# with 10.6 GU and sample 1# with 10.5 GU. Figure 4 depicts a graph of the film’s gloss effect at a 60° incidence angle. The range results show that the brass powder content has the greatest effect on the coating’s gloss, while the coupling agent content, coupling agent type, and pH of the solution during the modification process have less effect on the coating’s gloss. According to the results of the ANOVA of the gloss in Table 9, it was found that the ANOVA results of the above four factors were consistent with the results of the range, and the effect of brass powder content on the coating’s gloss was significant. Combined with the results of the ANOVA, the better process parameters for the preparation of brass powder water-based coating were determined to be: silane coupling agent KH560, silane coupling agent content of 6%, pH value of the solution during the modification process of 4.0, and brass powder content of 20%.

#### 3.2.2. Color Difference Analysis

**Figure 5:**
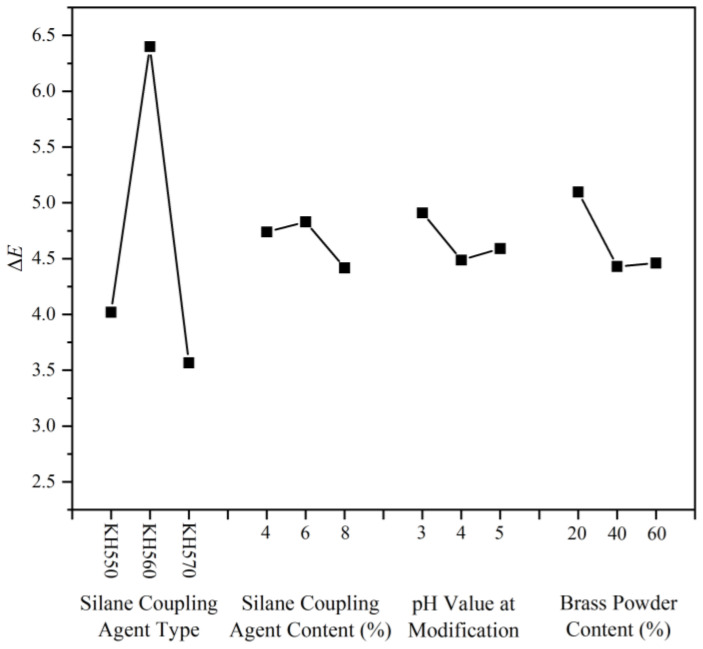
Effect of the color difference.

The primary factor used to determine whether the coating’s color is uniform or not is its color difference value, and the results of the calculation of this parameter are displayed in Table 10. Table 11 shows the visual analysis of color difference. According to the color difference effect (Figure 5) and the color difference ANOVA (Table 12), it is the coupling agent type that has a significant effect on the coating color difference value compared to the brass powder content, coupling agent content, and pH of the solution during modification. Combined with the results of the range, the prepared brass powder water-based coating has a more uniform coating color under the conditions of silane coupling agent KH570, silane coupling agent content of 8%, pH value of the solution during the modification process of 4.0, and brass powder content of 40%.

#### 3.2.3. Chromaticity Variation Analysis

**Figure 6:**
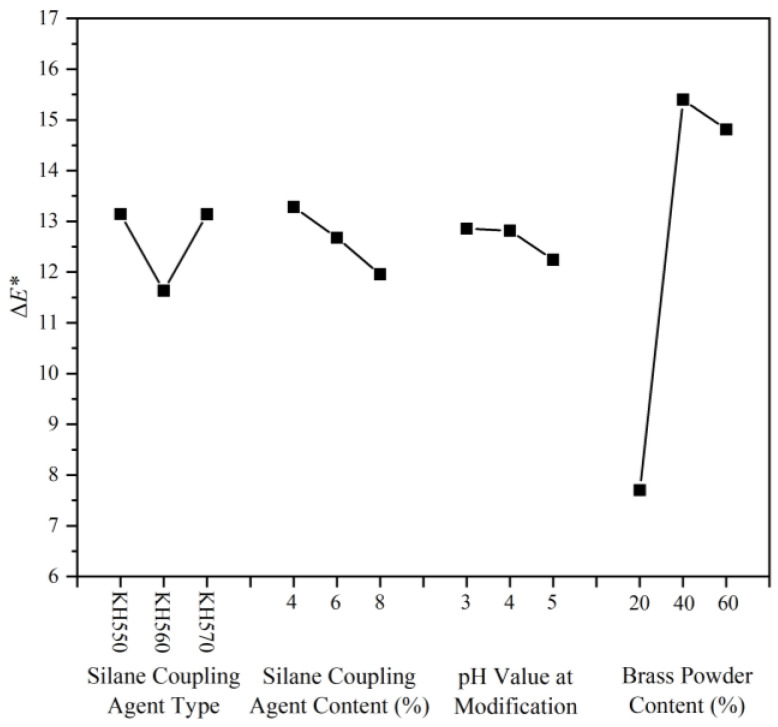
Effect of the chromaticity variation.

The chromaticity variation, i.e., the color change between the brass powder coatings and a pure water-based coating, can reflect the decorative color effect of the coating. The calculated results and visual analysis of the chromaticity variation of the coating are shown in Table 13 and Table 14. Compared with other samples, the chromaticity parameter of sample 7# was the best, reaching 16.15, followed by samples 2 and 8, with 16.12 and 15.57, respectively. The chromaticity variation effect graph (Figure 6) and the chromaticity variation ANOVA table (Table 15) showed that among the coupling agent type, coupling agent concentration, pH value of the solution during coating modification, and brass powder content, brass powder content is the one that can significantly affect the chromaticity variation. Combined with the results of the range, the highest chromaticity variation of the prepared brass powder water-based coatings was obtained under the conditions of silane coupling agent KH550, silane coupling agent content of 4%, the pH value of the solution during the coating modification process of 4.0, and brass powder content of 40%.

### 3.3. Analysis of Single-Factor Experiment Results

#### 3.3.1. Optical Performance Analysis

##### Gloss

**Figure 7:**
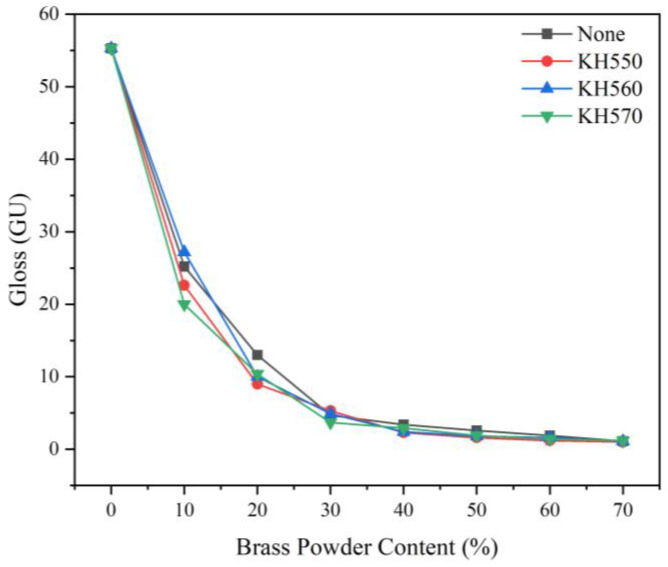
Gloss of different coupling agent-modified brass powder coatings with different brass powder content.

Table 16 and Figure 7 display the results of various coupling agent modifications on the gloss of the brass powder coatings. There is no discernible variation in gloss between unmodified and brass powder coatings modified by the three coupling agents, and the different modification treatments have no discernible impact on the gloss performance of the coatings. The gloss at incidence angles of 20°, 60°, and 85° revealed a negative association with the brass powder content, i.e., the higher the brass powder’s concentration, the lower the coating’s gloss, for both the unmodified and the brass powder coatings modified by the three coupling agents. This is so because brass powder is a solid powder, and adding more of it to the coating will make the coating surface rougher, which will reduce the coating’s gloss due to diffuse light reflection [56]. The gloss of the coating will differ greatly depending on the brass powder percentage, which should be between 0 and 10%. The weakening effect of brass powder concentration on the gloss of the coating gradually reduces in the 20% to 50% range. The gloss curve trend tends to level out as the brass powder percentage reaches 50%, and at this point, the brass powder content begins to saturate. Therefore, the brass powder concentration in the modified coating should be kept to 0–10% in order to provide a nice gloss effect. And within this range, the gloss performance of the brass powder coating following the KH560 treatment (sample 24#) is best.

##### Color Difference

**Figure 8:**
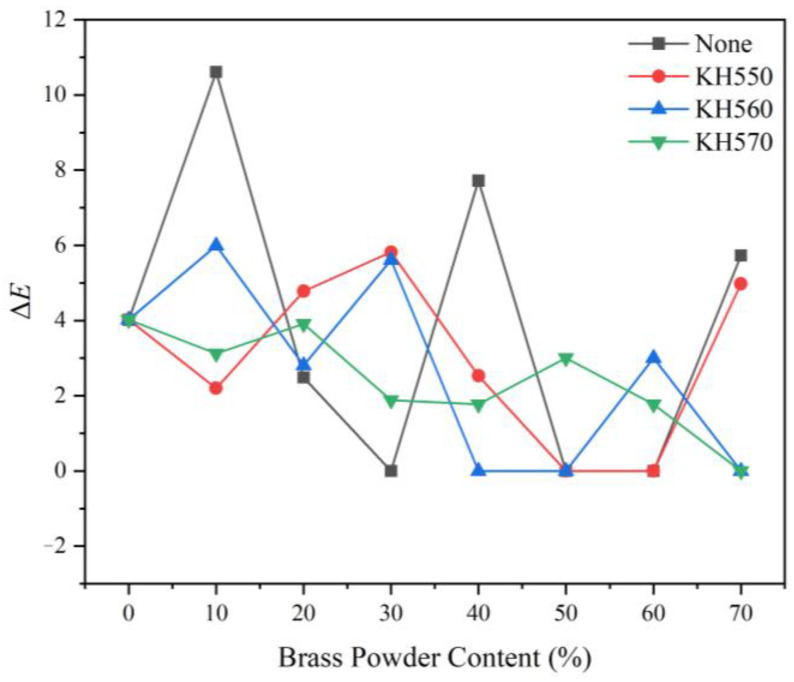
The color difference of different coupling agent-modified brass powder coatings with different addition contents.

Figure 8 illustrates the impact of various coupling agent modifications on the brass powder coating’s color difference. The color difference of the unmodified brass powder coating has the biggest change with an increase in the amount of brass powder. The coupling agent molecule has the structural formula Y-R-Si-X_3_, where Y in KH550, KH560, and KH570 is an amino functional group, an epoxy hydrocarbon functional group, and a methacryloyloxyalkyl organic functional group, respectively. R is a carbon chain with saturated or unsaturated bonds, and X is easily combined with silicon atoms and can be hydrolyzed by chlorine, methoxy, ethoxy, acetoxy, and other groups. The brass powder is modified using a silane coupling agent to produce silicon hydroxyl (Si-OH), which is then further dehydrated and condensed between coupling agent molecules to produce an oligomeric siloxane that contains several Si-OH groups. The -OH on the surface of the brass powder combines with the Si-OH in the oligomeric siloxane to produce covalent or hydrogen bonds [57]. The organic functional groups of the polymer chemically react with the Y group. Brass powder is hydrophobic and has a low affinity for water-based coatings without surface treatment. It is essentially insoluble in water and forms huge aggregates in water-based coatings, making it challenging to distribute evenly. Although some agglomeration of the silane-modified brass powder is unavoidable, due to the surface’s abundance of hydrophilic groups and its attraction for water-based coatings, the dispersion can be enhanced. Brass powder content does not significantly affect the coating’s color fluctuation. The best dispersion at 10% brass powder concentration is found with KH560 (sample 24#), followed by KH570 (sample 31#) and KH550 (sample 17#), and the coatings produced have a more uniform color.

##### Chromaticity Variation

**Figure 9:**
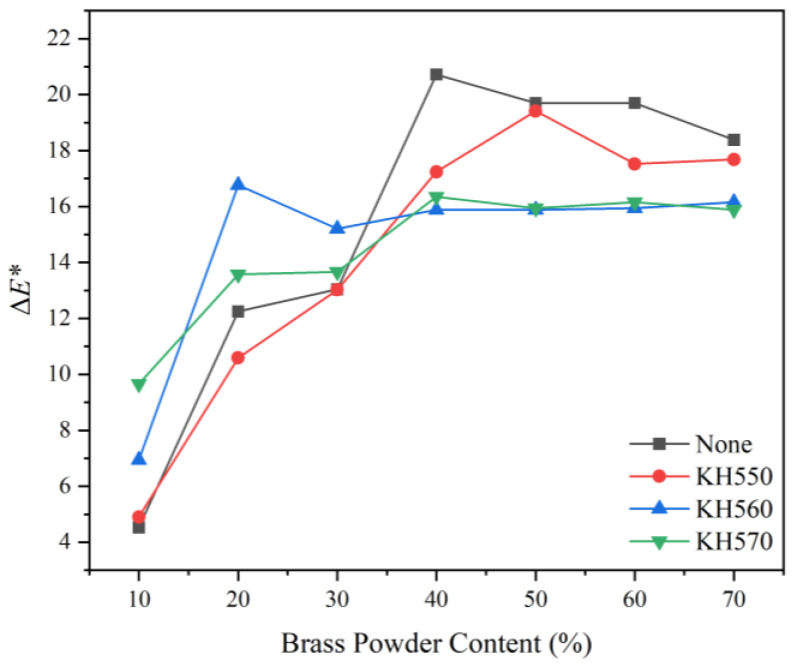
Variation curve of chromaticity variation of different coupling agent-modified brass powder coatings with different addition contents.

Figure 9 depicts the impact of various coupling agent modifications on the chromaticity variation of brass powder coatings. The chromaticity variation between brass powder coatings modified by the three coupling agents is small when the brass powder concentration is the same, and the modification treatment of using different coupling agents has little effect on the coating’s chromaticity variation. The chromaticity variation of unmodified and modified brass powder coatings with various coupling agents grew more pronounced with the increase in brass powder content. A brass powder content range of 10–40% was where the coatings’ chromaticity variation was the most pronounced. Because the brass powder can essentially lie flat on the surface of the Basswood substrate once the brass powder content exceeds 40%, the chromaticity variation of the coating tends to level off after that point. Moreover, further increases in the brass powder content will have little impact on the coating’s overall color. The most noticeable chromaticity difference with 10% brass powder content is found with KH560 (sample 24#), followed by KH570 (sample 31#) and KH550 (sample 17#).

##### Visible Light Reflectance

**Figure 10:**
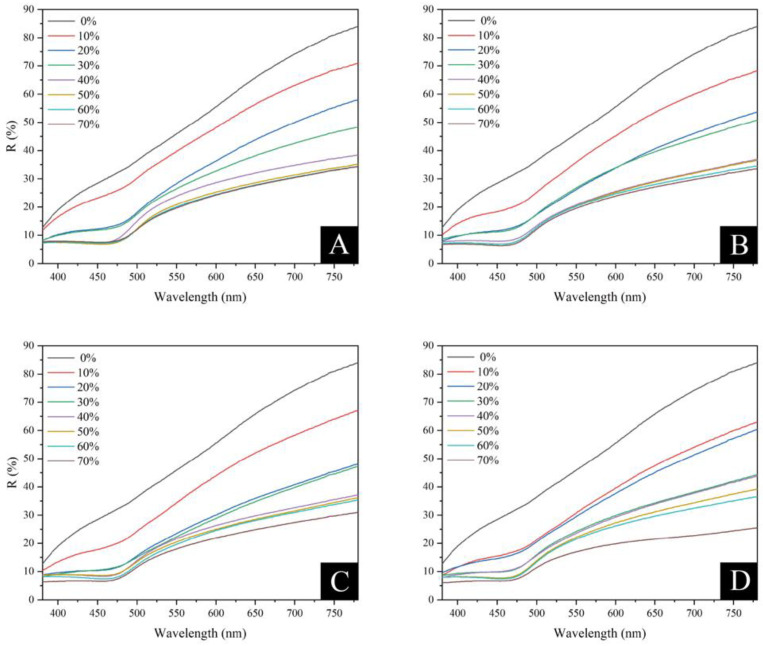
Visible wavelength reflectance of different coupling agent-modified brass powder coatings at different addition contents: (**A**) unmodified, (**B**) KH550 modified, (**C**) KH560 modified, (**D**) KH570 modified.

**Figure 11:**
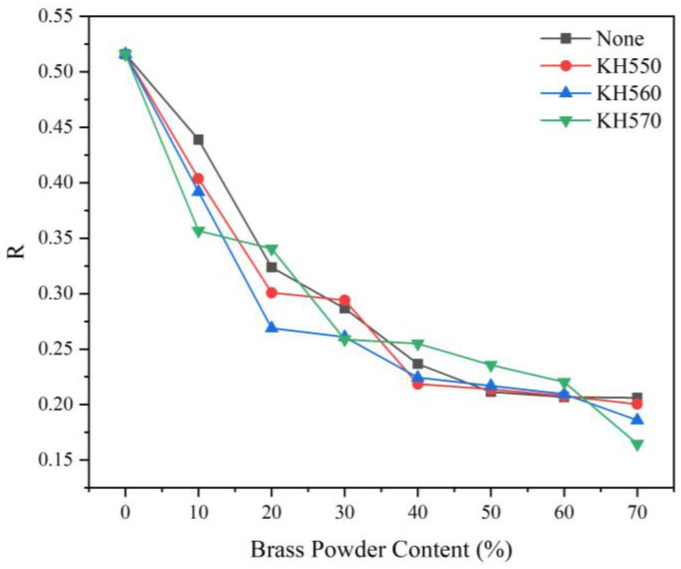
Visible wavelengths reflectance of different coupling agent-modified brass powder coatings with different brass content.

When the outside temperature is high, a coating with high reflectivity applied to the surface of wood can prevent heat from penetrating to the interior of the wood, thereby preventing heat aging and other issues while extending the service life of wooden objects. The reflectance of the visible wavelength band for various coupling agent-modified brass powder coatings with various brass content is shown in Figure 10, Figure 11, and Table 17. The R-value of visible wavelength reflectance of the brass powder coating was not significantly affected by the silane coupling agent treatment. Brass powder concentration and visual reflectance R-value had a negative relationship. The R-value in the 0 to 40% range of brass powder composition significantly declines as the brass powder content rises. The R-value remained relatively unchanged once the brass powder percentage reached more than 40%. The coatings display the greatest visible light reflectance at a brass powder concentration of 10%.

Figure 10 illustrates the coating samples’ color saturation. The color saturation of the sample increases and decreases with the difference between the high and low points of the reflectance spectrum. More bright colors result from higher saturation levels, while darker colors result from lower saturation levels. The color saturation of the coated samples steadily decreased with the rise in brass powder content, and the color gradually became duller. This tendency was seen in both the unmodified and modified brass powder coatings. Among them, the coating’s color brightness with a 10% brass powder concentration was the best.

The primary wavelengths of modified brass powder paint films are shown in Table 18. The primary wavelength of the coating color can be utilized as a starting point for assessing the coating samples’ overall visual color. All samples’ primary color wavelengths fell within the range of 586–590 nm. The wavelength of the yellow component of visible light ranges from approximately 565 to 590 nm; the closer the wavelength is to 590 nm, the more orange the sample seems overall, and the closer it is to 565 nm, the greener it appears overall. The main wavelength of the coating is closer to 586 nm as the brass powder content rises, demonstrating that the coating’s overall color is becoming more and more yellow, and that brass powder has a more pronounced effect on concealing the color and texture of the wood. These findings are in line with the findings of the chromaticity variation evaluation index.

#### 3.3.2. Mechanical Performance Analysis

##### Hardness

According to Table 19, the amount of various silane coupling agent-modified brass powders in the coatings affected how hard the Basswood surface’s coatings were. Due to the water-based coating’s inherent softness, Basswood surface coatings often have a low hardness. The coating’s hardness was 4B without the presence of brass powder. Because the thermoplastic acrylic self-drying technique primarily relied on self-crosslinking to generate a film, which is a physical film formation, its hardness performance was inferior. The film’s hardness rose sharply to HB when 10% brass powder was added. The hardness of the film steadily rose as the amount of brass powder increased, but the difference was not substantial. The silane coupling agent greatly increased the coating’s hardness compared to the unaltered brass powder coating. This is due to the fact that the Y group can interact with the resin matrix and serve as a coupling agent, improving the bonding efficiency of the resin and brass powder and significantly enhancing the mechanical qualities of the brass powder-resin system. The effect of the silane coupling agent’s type on the coating’s hardness is essentially the same when the coating contains 10–30% brass powder. When the coating contains 40% brass powder, the coating’s hardness after being altered by various coupling agents appears to vary. Due to the cross-linking principle, the epoxy functional group of KH560 and the carboxyl group of water-based acrylic acid underwent an addition reaction to form a chemical bond, whereas the amino functional group of KH550 and the carboxyl group of water-based acrylic acid underwent a coordination reaction to form an ionic bond [58]. The hardness of the brass powder coating modified by KH560 and KH570 was 3H, which was higher than that of the brass powder coating modified by KH550. The lower hardness is caused by the readily broken ionic bonds.

##### Impact Resistance

The amount of brass powder in the coating modified with various silane coupling agents affects the impact resistance of the Basswood surface coating according to Table 20. The amount of brass powder applied affects the coating’s impact resistance, whereas the silane coupling agent’s type and the amount of brass powder added both have an impact on the coating’s impact strength. The modified brass powder coating has a much higher impact strength than the unmodified brass powder coating. The unmodified brass powder coating has a maximum impact strength of 5 kg·cm in the 0 to 70% addition range, while the modified brass powder coating has a maximum impact strength of 8 kg·cm. This is since, following the treatment of the brass powder, the coupling agent molecules can create a molecular layer that is covalently bound to the surface of the brass powder, and that layer can significantly hinder water infiltration. The resin matrix strength will diminish as a result of the water intrusion, and the bond between the resin matrix and the interface will eventually fail, drastically reducing the mechanical capabilities of the composite material. The impact strength of the unmodified and modified brass powder coatings tended to rise and then fall with the brass powder content. The coating’s impact strength is optimum at 40% brass powder content for both unmodified and KH550-modified coatings. At a brass powder percentage of 50%, the KH560- and KH570-modified brass powder coatings showed the highest impact strength. Due to its double bonds, which boost strength, KH570 has the strongest impact resistance [59]. By reducing the freedom of the molecular chain and enhancing the impact resistance of the brass powder coating, the cross-linking reaction with the resin matrix increases the crosslinking density of the brass powder-acrylic resin.

##### Adhesion

According to Table 21, the amount of brass powder in coatings adjusted with various silane coupling agents affected how well they adhered to the surface of the Basswood. The adherence of the modified brass powder coating exhibits a more pronounced and significant improvement when compared to the unmodified brass powder coating. This is because of the coupling action of the silane coupling agent molecules with the covalently bonded molecular layer on the surface of the brass powder. The Y group resin molecules also effectively reduce the degradation of adhesion due to moisture intrusion and maintain or noticeably improve the mechanical properties of the composite [60]. Brass powder content and coating adhesion are inversely connected. The higher the brass powder concentration, the lower the coating adhesion. This is due to the fact that the coating’s adhesion is also influenced by its own characteristics and how well it adheres to the Basswood substrate. The internal dense structure of the coating is damaged when the brass powder is mixed into the water-based acrylic coating. As a result, the coating’s cohesion decreases, which is reflected in a decrease in the coating’s adhesion to the wood substrate [61]. Therefore, the coating that contains an excessive amount of brass powder doesn’t adhere as well. The coatings containing the modified brass powder showed the best adhesion (1 grade) when the content of metal fillers did not exceed 30%, which is consistent with the optimal samples (1 grade) in previous reports [62]. The coating modified by KH570 has the best adhesion of the three silane modifiers and can retain the adhesion of 2 grade even when the amount of brass powder is raised to 40%.

##### Roughness

According to Table 22, the amount of brass powder in coatings modified with various silane coupling agents affected how rough the Basswood surface coatings were. The brass powder-acrylic coating’s roughness was made worse by the coupling agent alteration. This is because the hydroxyl group reacts with the inorganic filler particles, causing the surface of the filler particles to progressively be replaced by the silane coupling agent condensate and making the particles’ surface rougher (Figure 2). As the amount of brass powder in the coating increased, the coating’s roughness frequently did as well. In the range of 0% to 10%, the growing trend was more pronounced. The lowest roughness of the coating was achieved when the content of metal filler was 10%, which was better than the roughness of 1.9 µm reported in reference [63].

#### 3.3.3. Aging Resistance Analysis

##### High-Temperature Accelerated Aging

**Figure 12:**
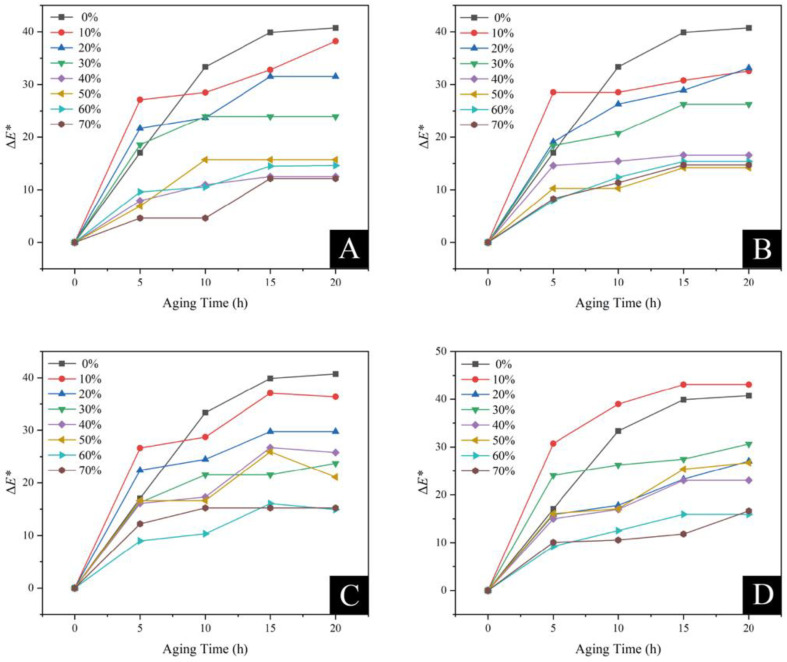
High-temperature accelerated aging resistance of different coupling agents-modified brass powder coatings at different addition contents: (**A**) unmodified, (**B**) KH550 modified, (**C**) KH560 modified, (**D**) KH570 modified.

Figure 12 depicts the chromaticity variation of the coating on Basswood before and after the high-temperature accelerated aging test with the different brass powder content adjusted by various coupling agents. Both the coupling agent-modified brass powder coatings and the unmodified brass powder coating produced chromaticity changes during the aging process. Due to the prolonged treatment that accelerated the aging of the Basswood and the coating, the chromaticity variation of the coating became increasingly apparent as the aging period rose. Within 0–5 h of the high-temperature accelerated aging treatment, the coating’s chromaticity variation was at its greatest. The brass powder concentration is roughly negatively connected with the chromaticity variation between the coating before and after aging throughout the same aging period, meaning that the higher the brass powder content, the less noticeable the coating’s chromaticity variation after high-temperature accelerated aging. This is because the brass powder itself does not readily react chemically at high temperatures to alter the color. Additionally, a high concentration of brass powder coating on wood might cover the wood itself during a high-temperature carbonization reaction since the material’s texture and color coverage are stronger. Because both the water-based coating and the wood are susceptible to denaturation at high temperatures, the weaker the color coverage of the wood substrate, and the higher the transparent water-based coating content in the coating, the greater the chromaticity variation on the coating with low brass powder content. After accelerated aging at high temperatures for 20 h, the coating with 10% unmodified brass powder had the biggest chromaticity variation of 38.22, which was greater than the coating with brass powder modified by coupling agents. This is because the silane coupling agent can coat the brass powder particles and raise the interfacial heat resistance of the brass powder particles, which to some extent retards the aging of the wood substrate and water-based coating substrate. Due to the addition of some KH550 internal amino groups to the reaction during polymerization, which reduces the coupling agent’s strong alkalinity and increases its temperature resistance [64], the brass powder coating modified by KH550 shows the least amount of chromaticity variation after high-temperature aging.

##### UV Aging Resistance

**Figure 13:**
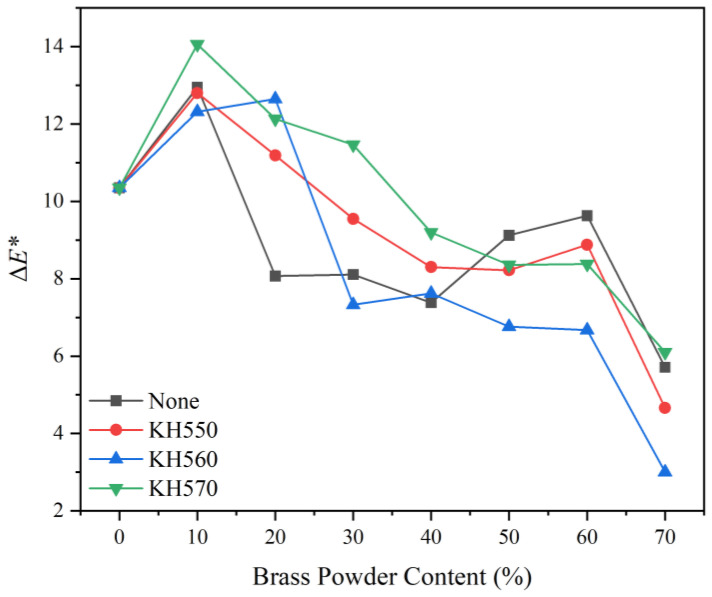
UV aging resistance of different coupling agent-modified brass powder coatings at different addition contents.

The high UV energy of sunlight, in conjunction with oxygen, readily initiates the polymer materials’ free radical chain photo-oxidative aging reaction. This results in the polymer macromolecular chain being broken or producing specific crosslinking, which directly affects the performance of polymer materials by causing color darkening, brittleness, hardening, surface cracking, and a decline in mechanical properties, among other aging phenomena, and eventually causes them to lose their value. Figure 13 depicts the chromaticity fluctuation of the Basswood surface coating with brass powder content in the coatings modified by various coupling agents. The amount of brass powder present and the coating’s UV aging chromaticity variation are negatively correlated. The less noticeable the chromaticity variation of the coating after UV aging, the higher the concentration of brass powder filler. The coating color is primarily influenced by UV light when the brass powder content is between 10% and 20%. This is because the effect of UV aging on brass powder is less pronounced than it is on polymeric water-based coatings, and the coating’s chromaticity fluctuation is more visible, the larger the proportion of water-based coatings in the coating. After the UV aging experiment, the unmodified brass powder coating had a more significant chromaticity variation. Brass powder coatings can be treated using silane coupling agents to increase their UV aging resistance. Among them, the coating modified with KH570 performed the best.

#### 3.3.4. Cold Liquid Resistance Analysis

One of the crucial criteria for testing the physicochemical qualities of wood coatings is the measurement of the coating’s liquid resistance. This parameter is based on a damage scenario induced by a liquid to which wooden objects may be exposed in daily life. The gloss of the Basswood surface coating after the cold liquid resistance test varied according to the amount of brass powder in the coating modified using various coupling agents, as indicated in Table 23. Following the evaluation of liquid resistance, all coatings displayed some degree of gloss degradation. At brass powder contents of 0 and 10%, the coatings’ loss of gloss was most noticeable. Detergent and coffee, out of the four liquids examined, had the biggest impact on the gloss of the brass powder coatings. Detergent and coffee had the greatest impact on the gloss of the brass powder coating modified by KH560 at 10% concentration (sample 24#), decreasing it by 20.5 and 11.3, respectively, while detergent and coffee had the least impact on the gloss of the brass powder coating modified by KH550 at 10% concentration (sample 17#), decreasing it by 9.9 and 8.0, respectively. KH570-modified brass powder coatings demonstrated more consistent gloss in the liquid resistance.

Table 24 illustrates the chromaticity variation of the coated surface of Basswood before and after the cold liquid resistance tests with various coupling agent-modified brass powder content. After the cold liquid resistance tests, the brass powder acrylic coating that had been altered using coupling agents had a significant change in coating color. The acrylic coating with brass powder responded the least to the four test liquids in terms of color change, whereas the KH560-modified coating was more sensitive to the effects of the test liquids and created more pronounced color changes. The coating’s chromaticity fluctuation was lowest at 10% brass powder content.

Table 25 displays the findings of the grading of the Basswood surface coatings following the measurement of liquid resistance. All of the coatings were essentially grade 1 for ethanol, leaving no mark on the coating film surface; grade 2 for coffee, leaving a minor discoloration mark on the coating film surface. The primary cause is that coffee is a dark brown color, which makes it easier for the coating to stain. The liquid resistance level for detergent is primarily grade 2, whereas the grade 3 performance of unmodified brass powder coating can be effectively improved by silane coupling agent modification. After the citric acid test, the brass powder coating modified by KH570 has improved liquid resistance and can still retain grade 2 liquid resistance even when the amount of brass powder increases to 50%. Based on the information in Table 25, silane coupling agent KH570 and a brass powder content of 10% resulted in the best liquid resistance performance of brass powder acrylic coating.

#### 3.3.5. Microscopic Morphology and Infrared Spectral Analysis of the Coating

**Figure 14:**
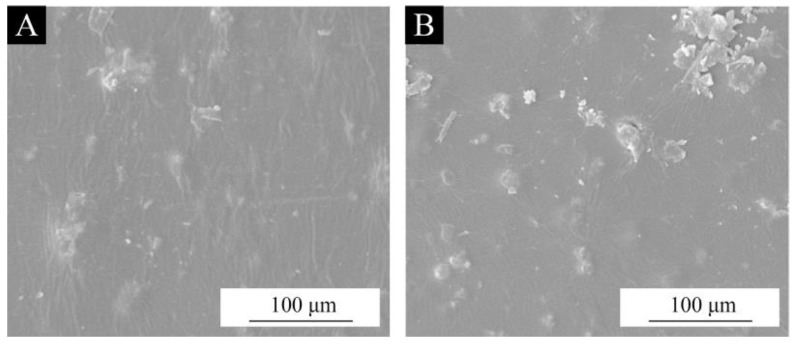
SEM images of Basswood surface films with 10% brass powder: (**A**) unmodified (Sample 10#), (**B**) KH570 modified (Sample 31#).

The brass powder-acrylic acid coating modified with 10% KH570 has the best overall performance, as can be seen from the findings above. Although the color difference is smaller, the chromaticity fluctuation is more noticeable, and the primary wavelength of color is closer to orange, its gloss is not significantly different from the pure brass powder water-based coating. It also has higher levels of hardness, impact resistance, and adherence. Its resistance to aging and fluids is also stronger. The microscopical characterizations of the brass powder-acrylic acid coatings with and without 10% KH570 modification are illustrated in Figure 14. The surface of the modified coating is essentially flat when compared to the pure brass powder water-based coating, and the minor protrusion is compatible with the findings of the roughness tests (Table 22). The material shows excellent surface condition.

**Figure 15:**
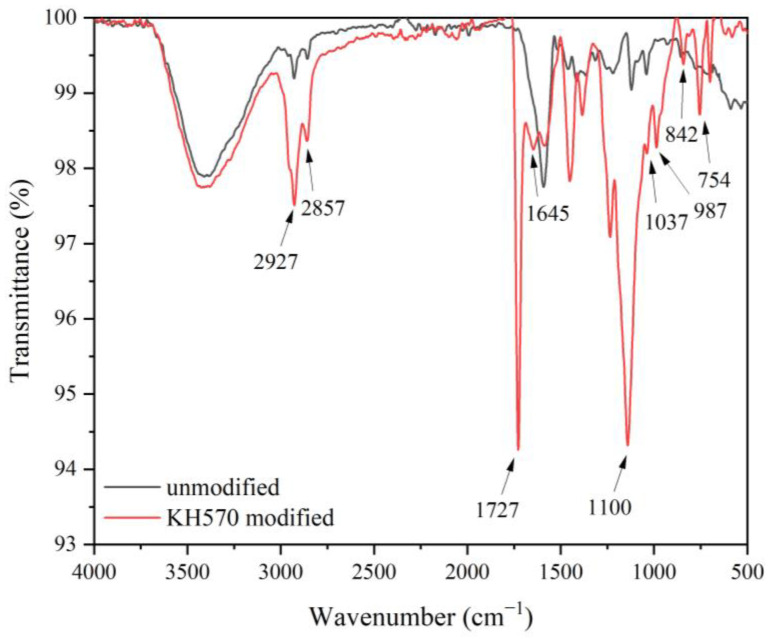
FTIR spectra of Basswood surface films with 10% brass powder.

Figure 15 shows the FTIR spectra of the brass powder-acrylic acid coating modified by 10% KH570 and the pristine brass powder-acrylic acid coating. The spectrum of the modified brass powder-acrylic acid coating clearly contains the characteristic peaks of KH570. The peaks for methylene are at 2927 cm^−1^ and 2857 cm^−1^. The Si-O-M (M = Cu, Zn, Si) asymmetric stretching mode is thought to be responsible for the strong adsorption band near 1100 cm^−1^ [65], demonstrating that a covalent link between KH570 and the brass powder was successfully formed. Si-C bonds have significant distinctive peaks at 842 cm^−1^ and 754 cm^−1^, and C-H in C=CH_2_ has an in-plane bending vibration peak at 987 cm^−1^. The asymmetric stretching vibration peak of silyl ether is 1037 cm^−1^. The deformation vibration peak of the C=O is at 1727 cm^−1^, and the deformation vibration peak of the C=C is at 1645 cm^−1^. The double bond is a significant element that can enhance the coating’s mechanical capabilities. The intensity of the C=C peak of the unmodified coating was greatly reduced in comparison to the FTIR spectra of the KH570-modified brass powder, and KH570 was successful in polymerizing acrylic acid.

## 4. Conclusions

Orthogonal tests were used to examine the effects of various factors (brass powder content, silane coupling agent type, silane coupling agent content, pH value) on the optical properties of modified brass powder-water-based acrylic coatings on a Basswood surface. The results showed that the coupling agent type and brass powder content had the greatest effects on the optical properties, with coating gloss, color difference, and chromaticity variation serving as the reference basis. Brass powder content had a negative correlation with coating gloss and a positive correlation with coating chromaticity variation under various silane coupling agent modifications. The silane coupling agent-modified coatings had a less pronounced color difference. The silane coupling agent can greatly increase the coating’s hardness, impact resistance, and adhesion in terms of mechanical properties. Aging resistance and liquid resistance tests reveal that the addition of a silane coupling agent can increase these properties. Combining all data, it is clear that the water-based acrylic acid coating containing 10% brass powder modified by KH570 (pH 5 of the solution at modification, coupling agent content 6%) has the best overall performance, improving the coating’s mechanical characteristics, aging resistance, and liquid resistance in addition to ensuring good optical properties, with a gloss of 20.0 GU, a color difference of 3.12, a hardness of HB, an impact resistance of 4 kg·cm, an adhesion of grade 1, and better liquid resistance and age resistance. These results provide the technological foundation for the development of artistic coatings for wood.

## Figures and Tables

**Table 1:** Dosage of brass powder modified with different silane coupling agent content.

Silane Coupling Agent Content (%)	Brass Powder (g)	Silane Coupling Agent (g)	Ethanol (g)	Deionized Water (g)
4	10	0.4	80	20
6	10	0.6	80	20
8	10	0.8	80	20

**Table 2:** Four factors and three levels of the orthogonal test.

Level	Silane Coupling Agent Type	Silane Coupling Agent Content (%)	pH Value at Modification	Brass Powder Content (%)
1	KH550	4	3	20
2	KH560	6	4	40
3	KH570	8	5	60

**Table 3:** Arrangement table of the orthogonal test.

Sample (#)	Silane Coupling Agent Type	Silane Coupling Agent Content (%)	pH Value at Modification	Brass Powder Content (%)
1	KH550	4	3	20
2	KH550	6	4	40
3	KH550	8	5	60
4	KH560	4	4	60
5	KH560	6	5	20
6	KH560	8	3	40
7	KH570	4	5	40
8	KH570	6	3	60
9	KH570	8	4	20

**Table 4:** Different silane coupling agent content modified copper powder acrylic paint film dosage details.

Sample (#)	Brass Powder Content (%)	Brass Powder Mass (g)	Water-Based Topcoat Mass (g)	Water-Based Primer Mass (g)
1	20	0.4	1.6	2.0
2	40	0.8	1.2	2.0
3	60	1.2	0.8	2.0
4	60	1.2	0.8	2.0
5	20	0.4	1.6	2.0
6	40	0.8	1.2	2.0
7	40	0.8	1.2	2.0
8	60	1.2	0.8	2.0
9	20	0.4	1.6	2.0

**Table 5:** Experimental arrangement of different amounts of silane coupling agent-modified copper powder on the comprehensive performance of the paint film.

Sample (#)	Silane Coupling Agent Type	Brass Powder Content (%)	Brass Powder Mass (g)	Water-Based Topcoat Mass (g)	Water-Based Primer Mass (g)
0	None	0	0	2.0	2.0
10		10	0.2	1.8	2.0
11		20	0.4	1.6	2.0
12		30	0.6	1.4	2.0
13		40	0.8	1.2	2.0
14		50	1.0	1.0	2.0
15		60	1.2	0.8	2.0
16		70	1.4	0.6	2.0
17	KH550	10	0.2	1.8	2.0
18		20	0.4	1.6	2.0
19		30	0.6	1.4	2.0
20		40	0.8	1.2	2.0
21		50	1.0	1.0	2.0
22		60	1.2	0.8	2.0
23		70	1.4	0.6	2.0
24	KH560	10	0.2	1.8	2.0
25		20	0.4	1.6	2.0
26		30	0.6	1.4	2.0
27		40	0.8	1.2	2.0
28		50	1.0	1.0	2.0
29		60	1.2	0.8	2.0
30		70	1.4	0.6	2.0
31	KH570	10	0.2	1.8	2.0
32		20	0.4	1.6	2.0
33		30	0.6	1.4	2.0
34		40	0.8	1.2	2.0
35		50	1.0	1.0	2.0
36		60	1.2	0.8	2.0
37		70	1.4	0.6	2.0

**Table 6:** Grading table of cold liquid resistance of the film.

Grade	Description
1	No changes: the test area is indistinguishable from adjacent areas.
2	Slight changes: the test area is hardly distinguishable from adjacent areas unless the light source is projected onto the test site revealing indications such as fading, discoloration, and discoloration. No change in the structure of the test surface, such as swelling, fiber protrusion, cracking, or blistering.
3	Moderate changes: visible in several directions, the test area is distinguishable from adjacent areas through indications such as discoloration, discoloration, and discoloration. No change in test surface structure, such as swelling, fiber protrusion, cracking, or blistering.
4	Visible changes: visible in all visible directions, the test area is clearly distinguished from adjacent areas through indications such as discoloration, discoloration and discoloration, and/or slight changes in the structure of the test surface, such as swelling, fiber protrusion, cracking, or blistering.
5	Severe changes: significant changes in test surface structure, and/or fading, discoloration and discoloration, and/or the surface material is removed in whole or in part, and/or filter paper is stuck to the surface.

**Table 7:** The gloss of brass powder coatings modified by different coupling agents.

Sample (#)	Gloss (GU)		
	20°	60°	85°
1	2.5	10.5	6.0
2	0.9	3.1	1.2
3	0.6	1.0	0.6
4	0.7	1.5	0.7
5	3.2	13.1	7.4
6	0.9	2.9	1.3
7	1.0	3.7	1.7
8	0.7	1.7	1.0
9	2.6	10.6	6.2

**Table 8:** Visual analysis table of gloss.

Sample (#)	Silane Coupling Agent Type	Silane Coupling Agent Content (%)	pH Value at Modification	Brass Powder Content (%)	Gloss (GU)
1	KH550	4	3	20	10.5
2	KH550	6	4	40	3.1
3	KH550	8	5	60	1.0
4	KH560	4	4	60	1.5
5	KH560	6	5	20	13.1
6	KH560	8	3	40	2.9
7	KH570	4	5	40	3.7
8	KH570	6	3	60	1.7
9	KH570	8	4	20	10.6
Mean 1	4.867	5.233	5.033	11.400	
Mean 2	5.833	5.967	5.067	3.233	
Mean 3	5.333	4.833	5.933	1.400	
Range	0.966	1.134	0.900	10.000	

**Table 9:** Analysis of variance (ANOVA) of gloss.

Factor	Sum of Squares of Deviations	Freedom	F Ratio	F Critical Value	Significance
Silane Coupling Agent Type	1.402	2	1.000	19.000	
Silane Coupling Agent Content	1.982	2	1.414	19.000	
pH Value at Modification	1.562	2	1.114	19.000	
Brass Powder Content	170.056	2	121.295	19.000	*
Error	1.40	2			

Note: * in Table 9 means significant.

**Table 10:** The color difference of brass powder coatings modified by different coupling agents.

Sample (#)	Chromaticity Parameter						Δ*E*
	*L*_1_	*a*_1_	*b*_1_	*L*_2_	*a*_2_	*b*_2_	
1	62.41	7.78	34.31	58.27	10.15	34.09	4.78
2	56.53	5.88	35.22	54.12	7.26	32.66	3.78
3	57.70	7.05	33.13	55.00	7.00	35.35	3.50
4	55.00	7.00	35.35	59.94	7.00	38.93	6.10
5	65.95	12.45	33.46	66.32	7.45	38.25	6.93
6	57.80	12.22	29.76	57.70	7.05	33.13	6.17
7	53.95	7.37	36.72	56.53	5.88	35.22	3.34
8	56.53	5.88	35.22	54.12	7.26	32.66	3.78
9	64.46	8.49	33.33	63.54	6.19	35.92	3.58

**Table 11:** Visual analysis table of the color difference.

Sample (#)	Silane Coupling Agent Type	Silane Coupling Agent Content (%)	pH Value at Modification	Brass Powder Content (%)	Gloss (GU)
1	KH550	4	3	20	4.78
2	KH550	6	4	40	3.78
3	KH550	8	5	60	3.50
4	KH560	4	4	60	6.10
5	KH560	6	5	20	6.93
6	KH560	8	3	40	6.17
7	KH570	4	5	40	3.34
8	KH570	6	3	60	3.78
9	KH570	8	4	20	3.58
Mean 1	4.020	4.740	4.910	5.097	
Mean 2	6.400	4.830	4.487	4.430	
Mean 3	3.567	4.417	4.590	4.460	
Range	2.833	0.413	0.423	0.667	

**Table 12:** ANOVA of the color difference.

Factor	Sum of Squares of Deviations	Freedom	F Ratio	F Critical Value	Significance
Silane Coupling Agent Type	13.898	2	49.110	19.000	*
Silane Coupling Agent Content	0.283	2	1.000	19.000	
pH Value at Modification	0.292	2	1.032	19.000	
Brass Powder Content	0.851	2	3.007	19.000	
Error	0.28	2			

Note: * in Table 12 means significant.

**Table 13:** The chromaticity variation of brass powder coatings modified by different coupling agents.

Sample (#)	Chromaticity Parameter			ΔE*
	L*	a*	b*	
0	70.42	8.93	30.96	-
1	61.89	8.55	34.01	9.07
2	54.49	7.54	33.02	16.12
3	56.73	6.75	34.21	14.24
4	57.34	7.46	37.33	14.63
5	65.51	9.98	34.82	6.34
6	56.57	9.38	32.31	13.93
7	55.24	6.63	35.97	16.15
8	55.33	6.57	33.94	15.57
9	64.12	7.13	34.99	7.70

**Table 14:** Visual analysis table of the chromaticity variation.

Sample (#)	Silane Coupling Agent Type	Silane Coupling Agent Content (%)	pH Value at Modification	Brass Powder Content (%)	ΔE*
1	KH550	4	3	20	9.07
2	KH550	6	4	40	16.12
3	KH550	8	5	60	14.24
4	KH560	4	4	60	14.63
5	KH560	6	5	20	6.34
6	KH560	8	3	40	13.93
7	KH570	4	5	40	16.15
8	KH570	6	3	60	15.57
9	KH570	8	4	20	7.70
Mean 1	4.020	4.740	4.910	5.097	
Mean 2	6.400	4.830	4.487	4.430	
Mean 3	3.567	4.417	4.590	4.460	
Range	2.833	0.413	0.423	0.667	

**Table 15:** Analysis of variance of the chromaticity variation.

Factor	Sum of Squares of Deviations	Freedom	F Ratio	F Critical Value	Significance
Silane Coupling Agent Type	4.550	2	6.445	19.000	
Silane Coupling Agent Content	2.646	2	3.748	19.000	
pH Value at Modification	0.706	2	1.000	19.000	
Brass Powder Content	110.135	2	155.999	19.000	*
Error	0.71	2			

Note: * in Table 15 means significant.

**Table 16:** Gloss of different coupling agent-modified brass powder coatings.

Sample (#)	Silane Coupling Agent Type	Brass Powder Content (%)	Gloss (GU)		
			20°	60°	85°
0	None	0	17.4	55.3	64.8
10		10	6.6	25.2	22.1
11		20	3.0	13.0	9.6
12		30	1.2	4.7	2.4
13		40	0.8	3.4	2.1
14		50	0.9	2.6	1.3
15		60	0.6	1.9	1.0
16		70	0.6	1.1	0.7
17	KH550	10	6.2	22.6	20.4
18		20	2.1	9.0	5.1
19		30	1.2	5.3	2.6
20		40	0.9	2.4	1.4
21		50	0.6	1.6	0.8
22		60	0.6	1.2	0.8
23		70	0.5	1.0	0.7
24	KH560	10	8.1	27.2	22.6
25		20	2.6	10.0	6.6
26		30	1.4	4.9	2.9
27		40	0.8	2.4	1.3
28		50	0.7	1.8	1.0
29		60	0.7	1.6	0.9
30		70	0.6	1.1	0.8
31	KH570	10	5.7	20.0	18.0
32		20	2.7	10.4	6.0
33		30	1.2	3.7	2.1
34		40	0.9	2.9	1.5
35		50	0.8	1.9	0.9
36		60	0.6	1.4	0.9
37		70	0.6	1.2	0.8

**Table 17:** Visible light reflectance of modified brass powder paint films with different coupling agent types with different brass content.

Brass Powder Content (%)	R			
	None	KH550	KH560	KH570
0	0.5158	0.5158	0.5158	0.5158
10	0.4389	0.4038	0.3919	0.3566
20	0.3238	0.3008	0.2688	0.3406
30	0.2867	0.2941	0.2610	0.2588
40	0.2367	0.2186	0.2245	0.2550
50	0.2115	0.2138	0.2169	0.2357
60	0.2068	0.2077	0.2094	0.2204
70	0.2062	0.2004	0.1860	0.1646

**Table 18:** Main wavelengths of modified brass powder paint films with different coupling agent types.

Brass Powder Content (%)	Main Wavelength			
	None	KH550	KH560	KH570
0	589.89	589.89	589.89	589.89
10	589.34	589.56	589.88	590.00
20	589.29	589.47	589.78	589.68
30	588.30	588.57	589.21	588.15
40	587.64	587.56	587.90	587.80
50	587.25	587.40	587.68	587.40
60	586.88	587.06	587.26	586.09
70	586.41	587.00	587.13	587.05

**Table 19:** The hardness of modified brass powder film with different coupling agent types.

Brass Powder Content (%)	Hardness			
	None	KH550	KH560	KH570
0	4B	4B	4B	4B
10	HB	HB	HB	HB
20	H	H	H	H
30	H	2H	2H	2H
40	H	2H	3H	3H
50	H	3H	3H	3H
60	H	3H	3H	3H
70	H	5H	5H	5H

**Table 20:** Impact resistance of modified brass powder films with different coupling agent types.

Brass Powder Content (%)	Impact Resistance (kg·cm)			
	None	KH550	KH560	KH570
0	1	1	1	1
10	1	2	3	4
20	3	3	4	4
30	3	3	5	5
40	5	8	6	7
50	4	7	8	8
60	3	6	7	7
70	4	8	5	6

**Table 21:** Different coupling agent types modified brass powder paint film adhesion.

Brass Powder Content (%)	Adhesion (Grade)			
	None	KH550	KH560	KH570
0	0	0	0	0
10	1	1	1	1
20	2	1	1	1
30	2	1	1	1
40	4	3	3	2
50	4	3	4	3
60	4	3	4	3
70	5	4	4	4

**Table 22:** The roughness of modified brass powder coating film with different coupling agent types.

Brass Powder Content (%)	Roughness (µm)			
	None	KH550	KH560	KH570
0	0.480	0.480	0.480	0.480
10	1.635	1.209	1.022	1.322
20	2.318	1.742	1.612	1.965
30	2.679	2.481	1.413	3.554
40	2.983	3.654	3.554	3.210
50	2.766	2.988	2.824	2.504
60	3.908	2.782	3.037	3.453
70	4.138	3.860	3.050	4.094

**Table 23:** Liquid resistance gloss of different coupling agent-modified brass powder coatings.

Sample (#)	Silane Coupling Agent Type	Brass Powder Content (%)	Gloss (GU)			
			Citric Acid	Ethanol	Detergent	Coffee
0	None	0	51.9	50.1	27.9	48.4
10		10	25.0	25.0	24.2	24.0
11		20	11.3	12.9	11.6	12.3
12		30	4.7	4.7	3.8	4.7
13		40	3.4	3.1	2.7	3.2
14		50	2.5	2.6	2.3	2.6
15		60	1.8	1.9	1.7	1.9
16		70	1.1	1.1	1.1	1.1
17	KH550	10	21.6	19.4	6.5	15.7
18		20	7.6	7.6	4.9	8.5
19		30	4.3	4.4	2.6	4.4
20		40	2.2	2.4	2.4	2.4
21		50	1.6	1.3	1.6	1.3
22		60	1.2	1.1	1.2	1.0
23		70	1.0	1.0	1.0	1.0
24	KH560	10	21.0	22.7	6.7	15.9
25		20	8.2	10.0	5.2	7.3
26		30	4.3	4.0	3.4	3.6
27		40	2.2	2.3	2.2	2.0
28		50	1.5	1.7	1.8	1.5
29		60	1.2	1.4	1.4	1.5
30		70	1.0	1.1	1.1	1.0
31	KH570	10	13.3	17.3	10.1	12.0
32		20	8.2	8.1	4.7	8.6
33		30	3.5	2.9	2.4	3.0
34		40	2.7	2.7	2.3	2.9
35		50	1.6	1.8	1.3	1.4
36		60	1.4	1.4	1.3	1.1
37		70	1.1	1.1	1.2	1.0

**Table 24:** Chromaticity variation of different coupling agent-modified brass powder coatings.

Sample (#)	Silane Coupling Agent Type	Brass Powder Content (%)	ΔE*			
			Citric Acid	Ethanol	Detergent	Coffee
0	None	0	2.89	1.51	2.89	2.61
10		10	5.46	5.21	4.04	5.19
11		20	6.19	1.25	4.98	2.01
12		30	3.15	2.67	3.23	8.11
13		40	5.33	3.86	12.74	5.39
14		50	15.51	0.00	9.62	9.02
15		60	15.51	0.00	9.59	9.02
16		70	8.91	5.43	18.48	5.71
17	KH550	10	4.49	1.93	9.54	4.22
18		20	11.19	2.17	11.19	6.34
19		30	10.75	4.60	9.08	7.31
20		40	15.22	8.62	8.48	10.04
21		50	16.30	8.22	7.96	12.83
22		60	15.94	2.54	9.39	9.01
23		70	13.46	2.49	7.34	8.41
24	KH560	10	2.97	4.57	2.97	5.12
25		20	13.72	1.50	11.27	7.28
26		30	13.89	2.80	7.35	7.07
27		40	14.77	9.19	11.82	10.56
28		50	14.94	9.19	14.77	10.08
29		60	20.39	1.50	11.93	10.35
30		70	15.23	3.00	15.23	3.69
31	KH570	10	6.64	4.99	5.75	8.80
32		20	1.27	4.51	4.61	3.07
33		30	5.79	1.17	3.38	6.75
34		40	8.33	2.54	2.98	2.98
35		50	6.05	7.32	9.38	6.05
36		60	21.34	2.95	6.84	9.23
37		70	18.12	0.00	11.82	5.87

**Table 25:** Liquid resistance grade of different coupling agent-modified brass powder coatings.

Sample (#)	Silane Coupling Agent Type	Brass Powder Content (%)	Liquid Resistance Grade (Grade)			
			Citric Acid	Ethanol	Detergent	Coffee
0	None	0	1	1	2	1
10		10	1	1	2	1
11		20	2	1	2	1
12		30	2	1	3	2
13		40	2	1	3	2
14		50	3	1	3	2
15		60	3	1	3	2
16		70	3	1	3	2
17	KH550	10	2	1	2	2
18		20	2	1	2	2
19		30	2	1	2	2
20		40	3	1	2	2
21		50	3	1	2	2
22		60	3	1	2	2
23		70	3	1	2	2
24	KH560	10	1	1	1	2
25		20	2	1	2	2
26		30	3	1	2	2
27		40	3	1	2	2
28		50	3	1	2	2
29		60	4	1	2	2
30		70	4	1	2	2
31	KH570	10	1	1	1	2
32		20	1	1	2	2
33		30	2	1	2	2
34		40	2	1	2	2
35		50	2	1	2	2
36		60	3	1	2	2
37		70	4	1	2	2

## Data Availability

The data presented in this study are available upon request from the corresponding author.
